# Food Waste to Energy: An Overview of Sustainable Approaches for Food Waste Management and Nutrient Recycling

**DOI:** 10.1155/2017/2370927

**Published:** 2017-02-14

**Authors:** Kunwar Paritosh, Sandeep K. Kushwaha, Monika Yadav, Nidhi Pareek, Aakash Chawade, Vivekanand Vivekanand

**Affiliations:** ^1^Centre for Energy and Environment, Malaviya National Institute of Technology, Jaipur, Rajasthan 302017, India; ^2^Department of Plant Breeding, Swedish University of Agricultural Sciences, P.O. Box 101, 230 53 Alnarp, Sweden; ^3^Department of Microbiology, School of Life Sciences, Central University of Rajasthan Bandarsindri, Kishangarh, Ajmer, Rajasthan 305801, India

## Abstract

Food wastage and its accumulation are becoming a critical problem around the globe due to continuous increase of the world population. The exponential growth in food waste is imposing serious threats to our society like environmental pollution, health risk, and scarcity of dumping land. There is an urgent need to take appropriate measures to reduce food waste burden by adopting standard management practices. Currently, various kinds of approaches are investigated in waste food processing and management for societal benefits and applications. Anaerobic digestion approach has appeared as one of the most ecofriendly and promising solutions for food wastes management, energy, and nutrient production, which can contribute to world's ever-increasing energy requirements. Here, we have briefly described and explored the different aspects of anaerobic biodegrading approaches for food waste, effects of cosubstrates, effect of environmental factors, contribution of microbial population, and available computational resources for food waste management researches.

## 1. Introduction

**Figure 1:**
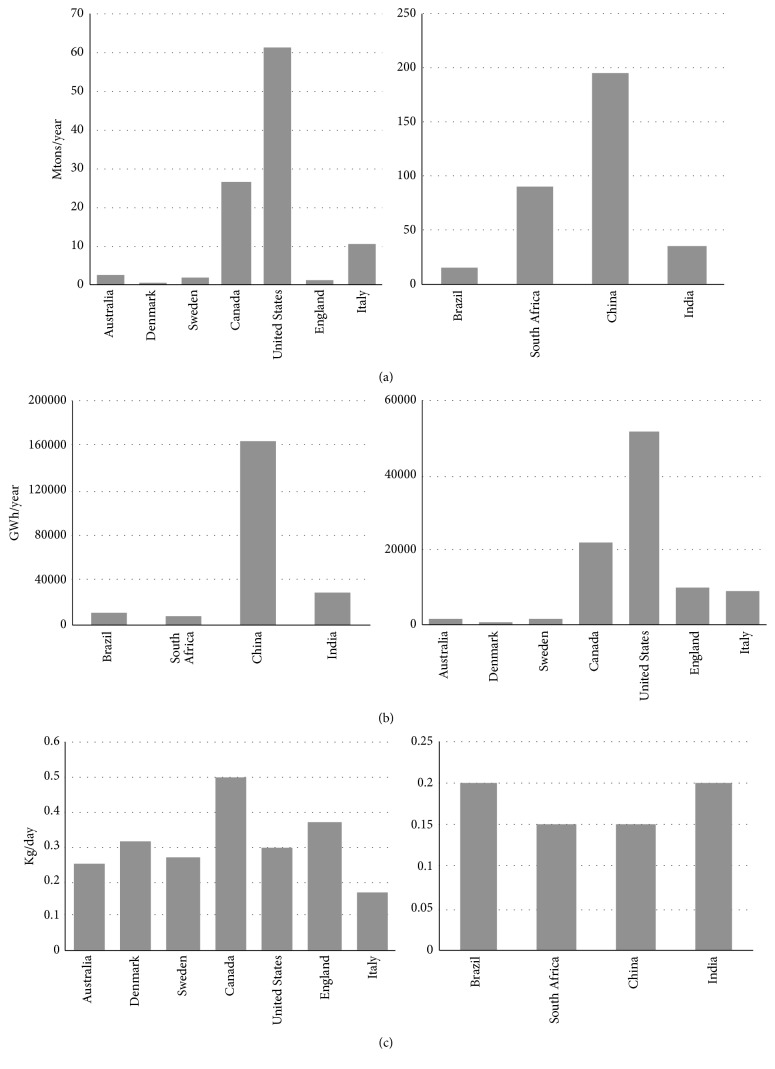
(a) Worldwide generation of food waste in developed and developing countries. (b) Worldwide bioenergy potential from FW in developed and developing countries. (c) Per capita food waste generation in developed and developing countries.

**Figure 2:**
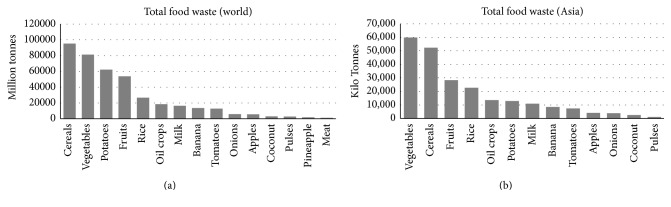
Typical wasted foods in world and in Asia.

*Food Waste*. Food waste (FW) (both precooked and leftover) is a biodegradable waste discharged from various sources including food processing industries, households, and hospitality sector. According to FAO, nearly 1.3 billion tonnes of food including fresh vegetables, fruits, meat, bakery, and dairy products are lost along the food supply chain [1]. The amount of FW has been projected to increase in the next 25 years due to economic and population growth, mainly in the Asian countries. It has been reported that the annual amount of urban FW in Asian countries could rise from 278 to 416 million tonnes from 2005 to 2025 [2]. Approximately 1.4 billion hectares of fertile land (28% of the world's agricultural area) is used annually to produce food that is lost or wasted. Apart from food and land resource wastage, the carbon footprint of food waste is estimated to contribute to the greenhouse gas (GHG) emissions by accumulating approximately 3.3 billion tonnes of CO_2_ into the atmosphere per year. Conventionally, this food waste, which is a component of municipal solid waste, is incinerated [3–7] or dumped in open area which may cause severe health and environmental issues. Incineration of food waste consisting high moisture content results in the release of dioxins [8] which may further lead to several environmental problems. Also, incineration reduces the economic value of the substrate as it hinders the recovery of nutrients and valuable chemical compounds from the incinerated substrate. Therefore, appropriate methods are required for the management of food waste [9]. Anaerobic digestion can be an alluring option to strengthen world's energy security by employing food waste to generate biogas while addressing waste management and nutrient recycling. The quantity of wasted food around the globe and its bioenergy potential via anaerobic digestion were reported earlier [10, 11] and are summarized in this work (Figures 1 and 2).

Food waste mainly consists of carbohydrates, proteins, lipids, and traces of inorganic compounds. The composition varies in accordance with the type of food waste and its constituents. Food waste consisting of rice and vegetables is abundant in carbohydrates while food waste consisting of meat and eggs has high quantity of proteins and lipids.  Table 1 summarizes the composition of food waste studied in different parts of the globe.

## 2. Anaerobic Digestion

Generation of methane via anaerobic process is an appropriate solution for food waste management. The process has lesser cost and low residual waste production and utilization of food waste as renewable source of energy [12, 13].  Table 2 summarizes the studies pertaining to anaerobic digestion of various kinds of FWs.

**Figure 3:**
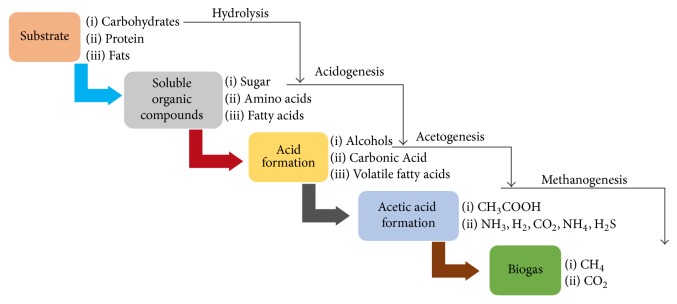
Anaerobic digestion phases.

Anaerobic digestion consists broadly of three phases, namely, enzymatic hydrolysis, acid formation, and gas production; Figure 3 depicts the digestion process.

### 2.1. Enzymatic Hydrolysis

In the first phase, large polymer molecules that cannot be transported to cell membranes by microorganisms are broken down by hydrolases secreted by facultative or obligate anaerobic hydrolytic bacteria. Hydrolysis breaks down the polymers into oligomer or monomeric units. Polysaccharides are broken down into oligosaccharides and monosaccharides; for example, (1) represents production of glucose molecules by starch hydrolysis. Proteins are broken down into peptides and amino acids and lipids are converted into glycerol and fatty acid.

(1) $$\begin{array}{c} nC_{6}H_{10}O_{5}+nH_{2}O⟶nC_{6}H_{12}O_{6} \end{array}$$

Mittal [42] reported that, in the anaerobic conditions, the hydrolysis rate is relatively slower than the rate of acid formation and depends on the nature of substrate, bacterial concentration, pH, and the temperature of the bioreactor. Other parameters such as size of the substrate particles, pH, production of enzymes, and adsorption of enzymes on the substrate particles also affect the hydrolysis rate. Bryant [43] reported that* Streptococcus* and* Enterobacter* are genera of anaerobes that are responsible for hydrolysis.

### 2.2. Acidogenesis Phase

In the second phase, acidogenesis takes place in which hydrolytic products are fermented to volatile fatty acids such as acetate, propionate, butyrate, valerate, and isobutyrate along with carbon dioxide, hydrogen, and ammonia. During acidification, facultative anaerobic bacteria utilize oxygen and carbon creating an anaerobic condition for methanogenesis. The monomers obtained in phase one become substrates for the microbes in phase two where the substrates are converted into organic acids by a group of bacteria.

Acetate, hydrogen, and carbon dioxide can be utilized directly for methane production. However, propionate, butyrate, valerate, and isobutyrate are introduced for further degradation by syntrophic acetogenic bacteria to form acetate and hydrogen [42–44].

### 2.3. Acetogenesis

Acetogenic bacteria belonging to genera* Syntrophomonas* and* Syntrophobacter* [44] convert the acid phase products into acetates (2) and hydrogen. Few acetate molecules are also generated by reduction of carbon dioxide using hydrogen as an electron source. Acetates will further be utilized by methanogens in subsequent steps. However, hydrogen released in the process exerts inhibitory effect on microorganisms. Therefore, in anaerobic digesters, acetogenic bacteria live in syntrophic relationship with hydrogenotrophic methanogens that remove the hydrogen by utilizing it for methane formation. Also, acetogenesis is the phase, which depicts the efficiency of the biogas production because 70% of methane arises when acetate reduces. Simultaneously, 11% hydrogen is also formed during the process [44].

(2) $$\begin{array}{c} nC_{6}H_{12}O_{6}⟶3nCH_{3}COOH \end{array}$$

### 2.4. Methanogenesis

In the last phase, methanogenesis takes place which is carried out by methanogens, belonging to Archaea. Methane can be produced either by fermentation of acetic acid or by reducing carbon dioxide. Therefore, the products of previous phase, that is, acetic acid, hydrogen, and carbon dioxide, act as a precursor for methane formation. Only 30% of methane produced in this process comes from carbon dioxide reduction carried out by methanogens [45, 46].

(3) $$\begin{array}{c} CH_{3}COOH⟶CH_{4}+CO_{2} \end{array}$$

Methane can be generated in two ways by two types of methanogens: (a) acetoclastic methanogens that produce methane from acetic acid and (b) hydrogenotrophic methanogens that utilize hydrogen to reduce carbon dioxide.

(4) $$\begin{array}{c} CO_{2}+4H_{2}⟶CH_{4}+3H_{2}O \end{array}$$

Table 3 summarizes genera active in anaerobic digestion and the microorganism cooperation in organic matter degradation.

## 3. Food Waste as a Substrate

Degradability of food waste used as substrate mainly depends upon its chemical composition. It is quite challenging to know the exact percentage of different components of the complex substrate because of its heterogeneous nature. Various researchers have investigated the potential of food waste as a substrate for biomethanation. Viturtia et al. [47] inspected two stages of anaerobic digestion of fruit and vegetable wastes and achieved 95.1% volatile solids (VS) conversion with a methane yield of 530 mL/g VS. In a study performed by Lee et al. [23], FW was converted into methane using a 5-L continuous digester, resulting in 70% VS conversion with a methane yield of 440 mL/g VS. Gunaseelan [24] used around 54 different types of food and reported methane yield ranged from 180 to 732 mL/g VS depending on the origin of wastes. Cho et al. [48] reported 472 ml/g VS methane yield with 86% anaerobic biodegradability of the Korean food waste. Yong et al. [49] have reported 0.392 m^3^ CH_4_/kg-VS when canteen food waste mixed with straw in the ratio of 5 : 1. Food waste as a substrate has potential to provide high biogas yield in comparison to cow manure, whey, pig manure, corn silage, and so forth [50].

## 4. Key Parameters Affecting Biomethanation

For anaerobes to work with high metabolic activity, it is imperative to have controlled environmental conditions. The methanogenic bacteria are very sensitive towards unfavorable survival conditions. Therefore, it is vital to maintain optimal condition to flourish the process of methanation. Biomethanation process primarily depends upon seeding, temperature, pH, carbon-nitrogen (C/N) ratio, volatile fatty acids (VFAs), organic loading rate (OLR), alkalinity, total volatile solids (VS), and hydraulic retention time (HRT) and nutrients concentration. It was also reported that the concentrations of water soluble material such as sugar, amino acids, protein, and minerals decrease and water nonsoluble materials such as lignin, cellulose, and hemicellulose increase in content [51].

### 4.1. Seeding

Seeding may speed up the stabilization of the digestion process. The most commonly used materials for inoculation are digested sludge from sewage plant, landfill soil, and cow dung slurry.

It was reported that the use of goat rumen fluid [52] as inoculum at the rate of 8% (v/v) is very efficient for biogas production.

### 4.2. Temperature

Methanogenesis has been reported from 2°C in marine sediments to over 100°C in geothermal areas [53]. Methanogens thrive best at around 35°C (mesophilic) and 55°C (thermophilic), respectively. Environmental temperature is also a huge concern for anaerobic microbial culture as change of acetic acid to methane depends mostly upon temperature. It has been reported that the optimum range of temperature is 35–40°C for mesophilic activity and 50–65°C for thermophilic activity [54, 55]. Bouallagui et al. [56] have reported that, at 4% total solid, methane content was found to be 58%, 65%, and 62% at temperatures 20°C, 35°C, and 55°C, respectively. At 8% total solid, methane content was found to be 57% and 59% at 35°C and 55°C, respectively. In a study reported by Kim et al. [57], methane content was found to be 65.6%, 66.2%, 67.4%, and 58.9% at temperatures 40°C, 45°C, 50°C, and 55°C, respectively. In another experiment performed by Gou et al. [58] codigestion of waste activated sludge with food waste was reported to have highest gas production rate at 55°C which was 1.6 and 1.3 times higher than the gas production at 35°C and 45°C.

### 4.3. pH

The pH of bioreactor affects the microbial activity in anaerobic digestion and its efficiency. Wang et al. [59] reported that optimum pH range is 6.3–7.8. Initially due to excess of carbon dioxide, pH drops to 6.2 and after 10 days it starts rising and stabilizes between 7 and 8. Also, Lee et al. [60] indicated that optimum range of methanogenesis using food waste leachate was 6.5–8.2. The main reasons for pH variation are VFAs, bicarbonate concentration, and alkalinity of the system. Goel et al. [61] used NaOH and NaHCO_3_ for controlling pH in anaerobic digestion used for biomethanation from food waste.

### 4.4. Carbon/Nitrogen Ratio

Mittal [62] has reported that digestion of substrate will proceed more rapidly if the C/N ratio would be 25–30 : 1. This leads to a conclusion that bacterial community use up carbon 25–30 times faster than nitrogen. If the ratio is not adequate, the nitrogen would get exhausted while there would be some carbon left which will cause bacteria to die. Excess of nitrogen would lead to ammonia formation which will inhibit the digestion process. Codigesting dairy manure, chicken manure, and wheat straw yielded maximum methane when C/N ratio was 27.2 with stable pH [59]. In another study performed by Zeshan et al. [63], anaerobic digestion performed well at C/N ratio of 27. An optimum amount of carbon content was having positive effect on avoiding excessive ammonia inhibition [64–66].  Table 4 [67] summarizes the C/N ratio of a few selected feed stock.

### 4.5. Volatile Fatty Acids (VFAs)

It has been reported that the production and accumulation of volatile fatty acid (VFAs) could show inhibitory and detrimental effects on anaerobic digestion process which could lead to slow production of biogas [68–70]. VFAs inhibition on the activity of methanogens is caused by a pH drop, which may lead to the activity loss of acid-sensitive enzymes [71]. Also, high levels of undissociated acids, which can penetrate cell membranes, may damage macromolecules [72]. The concentration of VFA in anaerobic digestion for the solid state of food wastes could rise up to 20 g/L, which is much higher than that in a wastewater anaerobic process [73]. In the optimum conditions required for metabolic activity, VFAs range in between 2000–3000 mg/L [74].

### 4.6. Organic Loading Rate (OLR)

Organic loading rate simply refers to quantity of feed processed per unit volume of reactor per day. Taiganides [75] had reported that controlled digestion is attained when the loading rate is between 0.5 kg and 2 kg of total VS/m^3^/d. In an experiment conducted by Nagao et al. [76], the volumetric biogas production rate increased to approximately 2.7, 4.2, 5.8, and 6.6 L/L/d as OLR increased to 3.7, 5.5, 7.4, and 9.2 kg-VS m^3^/d, respectively, and was maintained at the same. At the highest OLR (12.9 kg-VS m^3^/d), the volumetric gas production rate decreased below the gas production rate at OLR of 7.4 kg-VS m^3^/d. In a study performed for comparing autoclaved and untreated food waste [77], highest methane yield was obtained at organic loading rate of 3 kg VS/m^3^d for untreated food waste and at 4 kg VS/m^3^d for autoclaved food waste. The study was conducted at 2, 3, 4, and 6 kg VS/m^3^d. Agyeman and Tao [78] digested food waste with dairy manure anaerobically at different organic loading rates and reported that biogas production rate increased by 101–116% when OLR was increased from 1 to 2 g VS/L/d and only by 25–38% when OLR was further increased from 2 to 3 g VS/L/d. Specific methane yield peaked at the OLR of 2 g VS/L/d in the digesters with fine and medium food waste. Also, codigestion of food waste with activated sludge was performed in both mesophilic and thermophilic anaerobic systems at different OLR. The thermophilic system exhibited the best load bearing capacity at extremely high OLR of 7 g VS/L/d, while the mesophilic system showed the best process stability at low OLRs (<5 g VS/L/d) [79].

For a given size of biogas plant, there would be an optimum loading rate beyond which further loading will not be fruitful as it may lead to accumulation of excess VFAs and results in a collapsed reactor [80].

### 4.7. Ammonia

Biodegradation of protein or other nitrogen-rich substrate produces ammonia and exists in the form of ammonium ion (NH_4_^+^) and NH_3_ [81, 82]. It could be beneficial for the growth of microbes or sometime have detrimental effect on them [82, 83]. Ammonia plays a vital role in C/N ratio and could affect the performance of digestion process [59]. The reaction between ammonia and VFAs have been reported by Zhang et al. [84] and are as follows:

(5) CxHyCOOH⇌CxHyCOO−+H+

(6) NH3·H2O⇌NH4++OH−

(7) CxHyCOOH+NH3×H2O⟶CxHyCOO−+NH4++H2O,

where C_*x*_H_*y*_COOH represents VFAs.

### 4.8. Nutrient

Microbes use carbon to fulfil energy requirement and nitrogen for building cell wall structure. In addition, microbes also need small quantity of micro nutrient [85] such as calcium, sodium, potassium, magnesium, and chlorine. Also, for enzyme synthesis and for maintaining enzyme activity, heavy metal ions such as Cr, Co, Cu, Zn, and Ni are required in biomethanation [86–88]. Effect of concentration of sodium, potassium, and calcium was observed during anaerobic digestion activity. No inhibition was observed when concentration of calcium was increased up to 7000 mg/L [89]; however optimum concentration was reported 150–300 mg/L [90]. Concentration of heavy metals could have inhibitory effects on methanogenic activity and inhibition degrees depend upon many factors, such as the total metal concentration, chemical forms of the metals, pH, and redox potential [91, 92].

## 5. Biochemical Methane Potential (BMP)

Biochemical methane potential is an important assay for elucidation of anaerobic digestion. There is an increased adaptation of BMP assay in recent studies.

In an experiment performed for mixed food waste like boiled rice, cabbage, and cooked meat which were digested with cellulase as control has manifested greater rate of production of methane which is about 472 ml/g VS with total reduction in VS up to 86% [48].

In another study performed on canteen waste mixed with straw in different ratios, BMP for food waste and straw was 0.26 and 0.16 m^3^ CH_4_/kg-VS, respectively, which shows that food waste is easily biodegradable waste while the straw was difficult to degrade anaerobically which may be due to presence of lignin [49].

Digesting food and vegetables anaerobically yielded methane with a minimum amount of 0.3 L/g VS in every sample which also incorporate as commercial value for anaerobic digestion [93]. In an experiment conducted by Elbeshbishy et al. [94], preincubated seed sludge has been used along with running seed sludge for BMP test of food waste along with primary sludge. The maximum methane production rates using nonincubated inoculum were higher (114 mL CH_4_^−g^  TCOD_sub_) than those using preincubated inoculum at all substrate-to-inoculum ratios. Lisboa and Lansing [95] codigested four food waste substrates (meatball, chicken, cranberry, and ice cream processing wastes) for 69 days with flushed dairy manure and have reported an increase in methane production. Their findings suggested that addition of even small quantity of food waste to dairy manure has significantly enhanced the BMP levels. It was observed that the extent of increase in BMP following the addition of food waste had wide range starting from 67 to 2940% for ice cream and chicken processing waste, respectively.

Biogas potential of the dry fraction from pretreatment of food waste from households has been evaluated by Murto et al. [96]. A higher methane yield (152 ± 22 m^3^/ton) was obtained from digestion of the dry fraction alone. Dry fraction mixed with structural material produced lower levels of biogas (112 ± 21 m^3^/ton) compared to digestion of dry fraction alone.

Autoclaved and untreated food waste BMP assay was performed by Tampio et al. [77]. Food waste was autoclaved at 1600°C, 6.2 bar. It has been reported that methane yield at all the loading rates (2, 3, 4, and 6 kg-VS/m^3^/d) was 5–10% higher for untreated food waste which was 0.483 m^3^ CH_4_/kg VS as compared to 0.439 m^3^ CH_4_/kg VS obtained from autoclaved food waste.

## 6. Pretreatment Methods for Food Waste

Anaerobic digestion is now widely embraced to manage solid waste and energy recovery. However, the recalcitrance imposed by the compositional and structural features of food waste, that is, degree of polymerisation, crystallinity, lignin and pectin content, accessible surface area, and so forth, results in limiting the hydrolysis step of anaerobic digestion, such as food waste containing uncooked vegetables that consist of raw starch that has high degree of crystallinity which hinders its hydrolytic degradation. Therefore, a pretreatment step prior to anaerobic digestion is required to increase the degradability of food waste by increasing the surface area and reducing the degree of polymerisation and crystallinity. Pretreatment technologies like mechanical, thermal, chemical, and biological ones may be applied prior to anaerobic digestion to reduce the crystallinity and enhance the production of methane using wasted food. Research is earlier carried out to investigate the effect of different pretreatment methods on anaerobic digestion of food waste. Microwave pretreatment of food waste, with intensity of 7.8°C/min, resulted in enhanced biogas production and about 6% and 24% higher COD solubilisation [97]. In another study done by Izumi et al. [98], 28% higher biogas production was obtained using food waste when it is treated with beads mill. Ma et al. [99] has used different pretreatment techniques to kitchen waste. By adding HCL until the pH reduced to 2, 48% higher methane production was reported. Meanwhile heating the same food waste at 120°C at 1 bar for 30 minutes gave 24% higher gas production. Again, freezing the same food waste at −80°C for 6 h and thawing for 30 min result in 56% higher gas production. Applying pressure of 10 bar followed then by depressurization produces 48% more biogas.

Sometimes intensity of temperature plays a vital role in thermal pretreatment. Wang et al. [100] have reported that pretreating food waste at 70°C for 2 hours results in only 2.69% higher methane production while treating it for 1 hour at 150°C results in 11.9% higher gas production. Number of days of pretreatment could result in different rate of gas production. In an experiment conducted by Stabnikova et al. [101], total production of biogas increased up to 23.7% when freozen and thawed for 7 days. On the contrary, only mere increase was recorded when it was frozen and thawed for 12 days, that is, 8.5%. In a study, when food waste and fruit and vegetable waste heated at 175°C for 60 min this showed 7.9% and 11.9% decrease in biogas production, respectively [102]. Food waste (sorted from municipal solid waste) when treated with electroporation (400 pulses) [79] yielded 20–40% higher biogas production due to substrate cell breakage. Semiaerobic and anaerobic prehydrolysis of food waste resulted in 95% COD destruction with 500 ml/g VS methane yield [103]. Microaeration pretreatment [104] to the codigestion of brown water and food waste for 4 days with 37.5 ml O_2_/L/d has shown 21% higher methane yield for inoculated substrate whereas only 10% higher methane yield was observed for substrate without the inoculum.

## 7. Codigestion of Food Waste

Due to high potential for biomethanation, food waste is a reliable and promising substrate for anaerobic digestion activity. However, longer duration of digestion may sometimes lead to inhibition because of improper nutrient balance [105]. On the other hand, concentration of lipid in food waste is always higher than the limited concentration [84, 106]. To restrain this inhibition, many strategies have been adopted by researchers to codigest the food waste with cattle manure, green waste, or waste water sludge or with dairy waste.  Table 5 summarizes the codigestion of FW with other organic substrates for improving performance of anaerobic process.

## 8. Anaerobic Reactors

To carry out biomethanation in practical manner, different types of reactors are required. The researchers have used different types of anaerobic reactors such as single stage and two-stage reactors, semidry reactors, solid state anaerobic reactors, upflow anaerobic solid state reactors, and hybrid reactors for the execution of biomethanation process.

Configuration of process is quite important for efficiency of methane production process. For this, single stage and two-stage process have been employed for biodegradable waste treatment. Forster-Carneiro et al. [30] have reported that when all phases of anaerobic digestion, namely, hydrolysis, acidogenesis, acetogenesis, and methanogenesis, take place simultaneously in single reactor, system encounters fewer technical failures. When all polymeric compounds such as carbohydrates, protein, and fat are converted into CH_4_, H_2_S, NH_3_, and CO_2_ in a single vessel then that is termed as single stage reactor. Also, stability of single stage anaerobic digester for easily degradable FW is a matter of deep concern [23].

Chu et al. [107] reported that two-stage anaerobic digester has been used to produce both hydrogen and methane in two separate reactors from food waste. In this type of system, in the first stage, acidogens and hydrogen producing microorganisms which are having faster growth rate are enriched for hydrogen production and volatile fatty acid. In the second stage, acetogens and methanogens are built up where volatile fatty acids are converted into methane and carbon dioxide.

It has been reported that two-stage anaerobic digestion is providing more efficient operation as compared to single stage. Park and Li [65] have reported that highest methane recovery from kitchen waste when operated in both single stage and two-stage system was obtained as 90% (based on COD) which was determined at the OLR of 15 g COD/L/d. It was also reported by Massanet-Nicolau et al. [108] that methane yield from food waste increased by 37% in two-stage methane fermentation process. The highest methane yields from FWs were reported by Koike et al. [109]. Biogas production of 850 L/g VS during the two-stage hydrogen and methane production processing of FW was obtained by them.

On the other hand, solid state anaerobic digestion has several advantages over liquid anaerobic digestion in terms of smaller volume required for reactors, low material handling, low water requirement, and so forth. It has been reported that food waste is generally treated by liquid anaerobic digestion and organic fraction of municipal solid waste as well as lignocellulosic biomass can be treated by solid state anaerobic digestion [76].

Hybrid reactors also have been proposed by some researchers. In a study performed by Hai-Lou et al. [110], food waste was digested at 35°C for 16 days in a hybrid reactor. The result showed treatment efficiencies of 77–80% of total organic content removal, 59-60% volatile solid removal, and 79-80% total COD reduction were achieved. Also, high methane content (68–70%) from the methanogenic phase favors the application of hybrid anaerobic solid liquid bioreactor to practical solid waste management.

## 9. Mathematical Modelling of Anaerobic Digestion

Based on the total operating solid content, anaerobic digestion can be categorized as liquid state anaerobic digestion (L-AD) (TS ≤ 15%) or solid state anaerobic digestion (SS-AD) (TS ≥ 15%) [111]. L-AD is adopted to treat liquid organic waste such as sewage sludge, animal manure, and waste water from food processing unit while SS-AD is adopted to treat solid organic material such as yard trimmings, crop residues, and organic fraction of municipal solid waste and food waste [112, 113]. As compared to L-AD, SS-AD is having advantages of solid loading capacity, more volumetric biogas productivity, and less need of energy [114].

Besides having economic and environmental benefits and being a promising technology, a major disadvantage of solid state anaerobic digestion is the low rate of reaction [115, 116]. Slow release of soluble substrate for microbial metabolism could be the possible reason for this. Till date, SS-AD systems are operated empirically and still lacking in mechanistic tools for controlling the process [117]. Application of mathematical model can be applied to anaerobic digestion for mechanism explanation and its engineering process and parameters affecting biomethanation and their interaction with each other [34, 118]. Many researchers have adopted mathematical modelling for optimizing anaerobic digestion activity based on theoretical, empirical, and statistical approach. In a theoretical approach, six models were adopted, namely, two-particle model [119], reaction front [120, 121], distributed model [122–126], spatial temporal model [127, 128], modified ADM 1 [129–131], and diffusion limitation [132]. Empirical approach leads to logistic modelling [133] and general kinetic modelling [134, 135]. Also, statistical approaches such as linear regression and artificial neural network have been adopted [136–140].

Statistically derived models may emphasize prediction of system behaviour and are especially useful when there are a limited number of targeting outputs, while the models are black box models and might not provide enough information to unveil system mechanisms.

On the other hand, theoretical models provide more insight into the complex system mechanisms, while simplification is required to find general applications [34, 141].

## 10. Microbial Community Analysis

**Figure 4:**
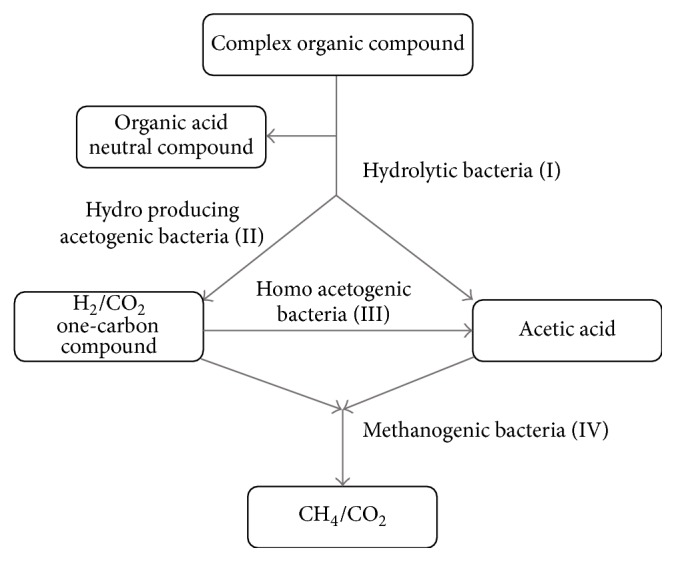
Significance of the microbial population in anaerobic digester.

Anaerobic digestion is the outcome of complex microbiome working in solidarity. A guild of microorganisms work on different phases of anaerobic digestion (Figure 4), maintaining a synergistic balance to ensure the stability of anaerobic digestion. However, anaerobic digesters often suffer with various instabilities pertaining to inhibition, foaming, and acidification especially at high organic load rates (OLRs) [142]. These instabilities are generally associated with the characteristics and dynamics of the microbial communities involved in anaerobic digestion process. Therefore, microbial community analysis, investigating the composition and behaviour of microbial communities, can be helpful to optimize stable and efficient process operation. The high-throughput sequencing technologies have further opened up new avenues for investigations of microbial communities during anaerobic digestion. Methods for revealing microbial community compositions are based on the generation of 16S rRNA gene clone libraries and 16S rRNA amplicons. Archaeal community are identified by targeting* mcrA* gene. The sequence reads are then analyzed by sophisticated bioinformatics tools for taxonomic distribution and functional annotations.

Lim and Wang, [104] studied microbial community for single phase and two-phase anaerobic digestion of food waste and found predominance of Firmicutes and greater bacterial diversity in two-phase continuous stirred tank reactor that led to 23% higher methane yield in comparison to single phase anaerobic digestion.* Methanosaeta* dominated the archaeal community of both single phase and two-phase reactors [143]. Cho et al. [144] investigated methanogenic community during dry anaerobic digestion of food waste and observed a significant reduction in methanogen diversity after acclimation to dry AD. Almost all sequences obtained from dry anaerobic digester sludge belonged to* Methanosarcina* genus reported to be more tolerant to sudden change in pH and use both acetoclastic and hydrogenotrophic pathways, which make them more suitable for surviving in comparison to* Methanosaeta*. Gou et al. [58] investigated effect of temperature and organic loading rate on microbial community of food waste anaerobic digestion and found significant effect of temperature on the richness of microbial community which was more diverse at 35°C in comparison to 45° and 55°C. At 55°C only 5 species remain abundant that explains that thermophilic bacteria are more sensitive towards temperature variation.

Wan et al. [145] classified the nucleotide sequences by using the ribosomal database project classifier software and showed that* Proteobacteria, Firmicutes, *and* Bacteroidetes* were the most abundant microorganisms during the entire process of anaerobic digestion. The diversity of microorganisms significantly increased during active methanogenesis in comparison to day 0, with addition of Synergistetes, Tenericutes, Spirochaetes, and Actinobacteria. Li et al. [142, 146] introduced disturbance in OLR into mesophilic anaerobic digester and carried out microbial community analysis during stable and deteriorative phases by employing pyrosequencing. Microbial communities were investigated using 454 pyrosequencing. Raw sequences were quality checked by mothur software and aligned with SILVA alignment. In his study, the acidogenic bacteria and syntrophic VFA oxidizers were found abundantly in deteriorative phase suggesting that, during high OLR, hydrolysis and acidogenesis surpassed the rate of methanogenesis which led to the irreversible acidification of accumulated VFAs.

Zamanzadeh et al. [147] investigated the effect of digestate recirculation on microbial community by using Illumina sequencing. Taxonomic assignment of sequences was done by using QIIME's uclust-based taxonomy assigner. Proteobacteria, Firmicutes, Chloroflexi, and Bacteroidetes were found to be the dominant bacterial phyla in both digester configuration types (with and without recirculation).* Methanosaeta *and* Methanobacterium *were dominant genera among archaeal population, accounting for 65% and 32% of* Euryarchaeota*'s reads in mesophilic digester without recirculation, while, in digester with digestate recirculation,* Methanosaeta *accounted for 91% of all* Euryarchaeota*'s. These results show the prevalence of acetoclastic methanogens over hydrogenotrophic methanogens, which acknowledge acetate reduction as the main pathway of methane formation. Similarly, Gulhane et al. [148] studied the microbial community under effect of no digestate recirculation, 25% digestate recirculation, and 100% digestate recirculation. Illumina sequenced reads were pair assembled using PANDAseq and mothur software was used to align and filter and trim and remove chimeras and classify and assign taxonomy. The result revealed the domination of hydrolytic and fermentative phyla in digester with no digestate recirculation, while syntrophic acetogenic bacteria dominated the digester with recirculation.

Guo et al. [149] carried out comparative analysis of the microbial community response to increasing OLR in mesophilic and thermophilic reactor and reported that mesophilic reactor had greater richness of microorganisms in comparison to thermophilic reactor. They also reported the dominance of* Methanosaeta* in archaeal community in mesophilic reactor while presence of* Methanothermobacter* and* Methanoculleus *were favored in thermophilic reactor.

## 11. Metagenomic Tool and Techniques for Advance Practices

In a fast-growing world, food wastage and its management are one of the major challenges faced by our society due to inherited high risk for human health and increasing environmental burdens. Strategic use of biodegradation processing on food waste can turn out into multiple societal benefits. Production of energy, that is, biogas through biomass of food waste, could be of major interest for easy storage and transport. Secondly, it reduces the hazardous effects on environment through the multiple layered food wastes processing and management. Production of soil additives and liquid fertilizers from organic food waste will be direct incentive from food waste management. Various 16S and 18S rRNA-based fragmented studies were performed through researcher for waste management treatment and microbial communities were identified from all the three taxonomic units of the microbial world, that is, Archaea, Bacteria, and Eukarya. In total, 4133 methanogenic bacteria were classified into Archaea* domain *and Crenarchaeota and* Euryarchaeota *are most visible group [33]. Methanogens have huge morphological diversity: cocci* (Methanococcus)*, Spirillaceae* (Methanospirillum)*,* Sarcina (Methanosarcina)*, rods* (Methanobacterium)*, short rods* (Methanobrevibacter)*, and filiforms* (Methanothrix)* [150]. Acetotrophic methanogens are the main obligatory anaerobes belonging to genus* Methanosarcina* which are involved in the processing of acetate to methane and carbon dioxide. Methanobacteriaceae family have been found associated with hydrogen binding methanogenic bacteria.* Methanosphaera stadtmaniae* and* Methanobrevibacter wolinii* are the two main groups of hydrogenotrophic microorganisms participating in anaerobic processing of fruit and vegetable [151].

The knowledge of the link between taxonomical and functional diversity and species richness can be a key for better understanding of ecosystem functioning in waste food treatment. Molecular methods like PCR, RFLP, microarrays, and sequencing have been utilized in the field of waste management. But these methods have own limitations for large scale functional characterization of ecological systems. Recently, captured metagenomics demonstrated the potential of functional characterization of microbial communities of agricultural soil on a large scale through NGS. Application of these approaches for food waste management can improve our understanding about treatment and enhance quality of treatment and management products [152]. Microbial communities can be used in more efficient manner in food waste management through exploration of available microbial resources and strategic use of available advance metagenomics practices.

### 11.1. Microarrays

Microarray is a one of the easiest and powerful tools to characterize differences in gene content between organisms and gene expression. Microarray technique has become popular due to large scale sequencing of microbial genomes year after year. Hundreds of microbial microarray based studies have been published such as 16S rRNA-based taxonomic microarray for Proteobacteria [153] and Alphaproteobacteria [154], Actinomycetes microarray [155], Bacillus-PhyloChip [156], Burkholderia-PhyloChip [157], ECC-PhyloChip [158], Compost Community-Microarray [159], Freshwater Sediment-Microarray [160], Soil microbial community PhyloChip [161], SRP-PhyloChip [162], and Nitrifier-Microarray [163]. Recent advancement in sequencing and molecular technologies has opened the doors for metagenomic studies. Captured metagenomics is one example for high resolution study for soil metagenomes [152, 164].

### 11.2. Next-Generation Sequencing

The high-throughput next-generation sequencing (HT-NGS) technologies produce a lot more data compared to capillary sequencing based method. Sequencing technology revolution started with Roche 454 GS FLX+ and currently it can produce relatively long read length (approx. 700 bp) and low number of reads (approx. 1 million reads/run) and is used for different applications such as examining 16S variable regions, targeted amplicon sequences, microbial genomes, BACs, and plastids. Illumina is one of the biggest players in the sequencing market with their versatile range of instruments and is ideal for genome sequencing and resequencing, transcriptome sequencing, SNP detection, and metagenomic studies. Illumina read length (50–300 bp) and read number (25 Million–6 billion per run) vary from platform to platform [165]. Ion Torrent technology (Ion PGM and Ion proton) is relatively new and semiconductor based sequencing platform. Potential of the platform varies with respect to the semiconductor chip that is used, that is, Ion 314™ Chip v2, Ion 316™ Chip v2, and Ion 318™ Chip v2 (read length: 200–400 bp, reads/run: 500K–5 million). It is used for various sequencing applications such as amplicons, small genomes, and targeted genomic sequencing. Automated workflow from sample preparation to analysis makes it ideal for smaller sized studies and routine practices [166]. PacBio RS have been developed for long read lengths through single molecule real-time sequencing technology, which can generate reads from 1 kb up to 60 Kb. Each SMRT cell can generate approx. 50,000 reads. Longer read length feature makes it ideal for sequencing small genomes such as bacteria or viruses, regions of high G/C content and DNA with modified bases (methylation, hydroxymethylation), resequencing projects and so forth [167].

### 11.3. Bioinformatics Resources

In 1970, Paulien Hogeweg and Ben Hesper coined the term “bioinformatics” for the study of information processes in biological systems as technique. Currently, bioinformatics is enormously integrated in almost all biological fields. The success of bioinformatics is mainly due to the recent advancements in computational resources and infrastructure across the globe which has facilitated bioinformatics research on complex biological systems [168].

Molecular insight in the diversity of the microbial communities is a relatively young field as not much was known about it prior to 1975 due to the unavailability of advance methods, tool, and techniques [169]. The advancement of sequencing and computational technology promoted metagenomics researches which increased the bioinformatics outreach in microbial informatics and experimentation. Further development of the bioinformatics methods resulted in a large number of databases, tools, and data formats for the analysis of microorganism and microbiome related studies which enhanced our knowledge and understanding of microbial populations [170]. In recent years, the advancement of high-throughput next-generation sequencing (NGS) platforms has enabled large scale sequencing efforts for the exploration of microbial ecosystems. Consequently, the microbial ecological analysis in the near future will need a paradigm shift from data generation to data management and sharing and hypothesis driven and targeted data generation [171], in silico generated knowledge coding, mining, and networking to improve our encoded models for new knowledge discovery [172, 173]. Here (Table 6) we have reviewed major microbial databases and tools that can be useful for microbial research application in emerging applied fields like food waste management and applications.

## 12. Conclusions

Proper disposal of food waste has posed a stern pecuniary and environmental concern. It appears that conversion of food waste into energy via anaerobic processes in terms of methane is economically viable. However, difficulties accompanying the collection as well as transportation of food waste should also be considered. Nevertheless, the stumpy or no cost of food waste along with the environmental aids considering the waste discarding would balance the initial high investment costs of the biorefineries. Moreover, the efficacy and cost base of the generation could be upgraded by intensifying research and optimization studies on assimilating different value-added product manufacturing processes.

## Figures and Tables

**Table 1:** Composition of FW reported in various literatures.

Moisture	Total solid	Volatile solid	Total sugar	Starch	Cellulose	Lipids	Protein	Ash	References
75.9	24.1	NR	42.3	29.3	NR	NR	3.9	1.3	[14]
80.3	19.7	95.4	59.8	NR	1.6	15.7	21.8	1.9	[15]
82.8	17.2	89.1	62.7	46.1	2.3	18.1	15.6	NR	[16]
75.2	24.8	NR	50.2	46.1	NR	18.1	15.6	2.3	[16]
85.7	14.3	98.2	42.3	28.3	NR	NR	17.8	NR	[17]
82.8	17.2	85.0	62.7	46.1	2.3	18.1	15.6	NR	[18]
61.3	38.7	NR	69.0	NR	NR	6.4	4.4	1.2	[19]
81.7	18.3	87.5	35.5	NR	NR	24.1	14.4	NR	[20]
81.5	18.5	94.1	55.0	24.0	16.9	14.0	16.9	5.9	[21]
81.9	14.3	98.2	48.3	42.3	NR	NR	17.8	NR	[22]

**Table 2:** Anaerobic digestion processes of food waste for methane production.

Waste	Inoculum	Vessel type	Duration (d)	HRT (d)	CH_4_ yield (ml/g VS)	% CH_4_	References
FW	Cow manure	Bioreactor with .5 L working volume	29	1	530	70	[23]
FW	Anaerobic SS	Pilot scale 5 tons/d	90	NR	440	70	[24]
FW	Anaerobic SS	Bioreactor with 12 L working volume	60	20	NR	68.8	[25]
FW	SS	Bioreactor with 4.5 L working volume	200	1–27	520	90	[26]
FW	NR	900 m^3^ tank volume	426	80	399	62	[27]
FW	Anaerobic SS	CSTR with 3 L working volume	225	16	455	NR	[28]
FW	NR	Digester with 800 ml working volume	30	Batch	410	66	[29]
FW	Anaerobic SS	Batch	28	10–28	440	73	[30]
FW	SS	CSTR with 10 L working volume	150	5	464	80	[31]
FW	Landfill soil and cow manure	Batch 5 L	60	20–60	220	NR	[32]

**Table 3:** Microorganism cooperation in organic matter degradation [33, 34].

Reaction Type	Microorganism	Active Genera	Product
Fermentation	Hydrolytic bacteria	*Bacteroides, Lactobacillus, Propionibacterium, Sphingomonas, Sporobacterium, Megasphaera, Bifidobacterium*	Simple sugars, peptides, fatty acids

Acidogenesis	Syntropic bacteria	*Ruminococcus, Paenibacillus, Clostridium*	Volatile fatty acids

Acetogenesis	Acetogenic bacteria	*Desulfovibrio, Aminobacterium, Acidaminococcus*	CH_3_COOH

Methanogenesis	Methanogens (Archaea)	*Methanosaeta, Methanolobus, Methanococcoides, Methanohalophilus, Methanosalsus, Methanohalobium, Halomethanococcus, Methanolacinia, Methanogenium, Methanoculleus*	CH_4_

**Table 4:** C/N ratio for some materials.

Material	% N	C : N
Animal urine	15–20	1
Cotton stalks	1.7	30
Cow, buffalo manure	1.4–3	15–40
Oat straw, flax straw	1–1.2	50–60
Wheat and rice straw	0.3–0.5	120–150
Sawdust	0.1–0.25	200–500

**Table 5:** Codigestion of food waste with other organic substrates.

Feedstock	Action of codigestion	Influencing factor	Ref.
FW + CM	Improve methane yield and system stability	High buffering capacity and trace elements supplement	[29]
FW + livestock waste	Improve methane yield and VS reduction	Higher buffering capacity	[35]
FW + yard waste	Improve methane yield	Less VFA accumulation	[36]
FW + dewatered sludge	Enhance system stability	Less inhibition from Na^+^	[28]
FW + sewage sludge	Afford high organic loading rate	High buffering capacity from ammonia	[37]
FW + green waste	Improve VS reduction	C/N ratio	[38]
FW + brown water	Improve methane yield	High buffering capacity	[39]
FW + press water	Improved system stability and methane yield	High buffering capacity	[40]
FW + distiller's grain	Improved biogas production	High buffering capacity from ammonia	[41]

**Table 6:** Bioinformatics tools and data bases used for microbial community analysis.

Database	Feature description	Web/open source	Availability
*Microbial genome and metagenomic data resource*			
*IMG*	Integrated Microbial Genomes and Microbiome. Repository of 33,116 genome datasets and 4,615 microbiome dataset	Yes/yes	https://img.jgi.doe.gov/
*MGDB*	Microbial genome database with 4742 genomes	Yes/yes	http://mbgd.genome.ad.jp/
ENSEMBL	Access to over 40,000 Bacterial Genomes	Yes/yes	http://bacteria.ensembl.org/index.html
RefSeq (microbial)	Archaeal and bacterial repository at NCBI Reference Sequence	Yes/yes	https://www.ncbi.nlm.nih.gov/refseq/

*Microarrays and gene expression database*			
(M3D)	Many Microbe Microarrays Database	Yes/yes	http://m3d.mssm.edu/
B*µ*G@Sbase	Microarray datasets for microbial gene expression	Yes/yes	http://bugs.sgul.ac.uk/bugsbase/tabs/experiment.php
*COLOMBOS *	Collection of bacterial gene expression compendium.	Yes/yes	http://www.colombos.net/
Microbeonline	Repository of 3707 genomes, gene expression data for 113 organisms	Yes/yes	http://www.microbesonline.org/

*Taxonomic, Functional Annotation and Comparative Genomics*			
POGO	Database of Pairwise-Comparisons Of Genomes and Orthologous genes	Yes/yes	http://pogo.ece.drexel.edu/about.php
*MicroScope*	Microbial Genome Annotation & Analysis Platform	Yes/yes	https://www.genoscope.cns.fr/agc/microscope/home/
*AGeS*:	A Software System for Microbial Genome Sequence Annotation	Yes/yes	http://www.bhsai.org/ages.html
NMPDR	National Microbial Pathogen Data Resource for annotation, comparative genomics with an emphasis on the food-borne pathogens	Yes/yes	http://www.nmpdr.org/FIG/wiki/view.cgi
*MetaPathways*:	A pipeline for taxonomic and functional annotation from environmental sequence information.	Yes/yes	http://hallam.microbiology.ubc.ca/MetaPathways/
*ShotgunFunctionalizeR*	an R-package for functional comparison of metagenomes.	Yes/yes	http://shotgun.math.chalmers.se/
*MG-RAST*	automated analysis platform for metagenomes based on sequence data	Yes/yes	http://metagenomics.anl.gov/
*MEGAN*	A comprehensive toolbox for interactively analyzing microbiome data	No/yes	http://ab.inf.uni-tuebingen.de/software/megan6/welcome/

*Metabolic analysis and modelling tool and databases*			
*CellDesigner*	Metabolic pathway reconstruction and simulation	Yes/yes	http://www.celldesigner.org/
*E-zyme*	Prediction of EC numbers from chemical transformation pattern	Yes/yes	http://www.genome.jp/tools/e-zyme/
*Triton*	Tool for Enzyme Engineering	Yes/yes	www.ncbr.muni.cz/triton/
*ECMDB*	*E. coli* Metabolme database	Yes/yes	http://ecmdb.ca/
*MicrobesFlux*:	A web platform for genome reconstruction and constraint-based modelling	Yes/yes	http://www.microbesflux.org/
*MetaCyc*	MetaCyc Metabolic Pathway Database	Yes/yes	http://metacyc.org/
*MetaBioMe*	Datamining engine for known Commercially Useful Enzymes (CUEs) in metagenomic datasets and genomes	Yes/yes	http://metasystems.riken.jp/metabiome/
*Metabolome Searcher*	HTS tool for metabolite identification and metabolic pathway mapping directly from mass spectrometry and metabolites		http://procyc.westcent.usu.edu/cgi-bin/MetaboSearcher.cgi
*ProCyc*	An open resource for the study of metabolic capabilities in microorganisms from food, environment, and specific pathogens from these sources.	Yes/yes	http://procyc.westcent.usu.edu:1555/
*MEMOSys*	Bioinformatics platform for genome-scale metabolic models	Yes/yes	http://icbi.at/software/memosys/memosys.shtml
EAWAG-BBD	Microbial biocatalytic reactions and biodegradation pathways	Yes/yes	http://eawag-bbd.ethz.ch/
Desharky	Microbial biodegradation to host metabolites.		http://soft.synth-bio.org/desharky.html
PathPred	Microbial biodegradation	Yes/yes	http://www.genome.jp/tools-bin/pathpred/pathpred.cgi
CRAFT	Chemical Reactivity and Fate tool Biodegradation of aerobic bacteria		https://www.mn-am.com/products/craft
EAWAG-BBD/PPS/BPT	Biodegradation information of aerobic/anaerobic bacteria	Yes/yes	http://eawag-bbd.ethz.ch/
MetaboleExpert	Biodegradation by plants and animals		http://www.compudrug.com/metabolexpert
ModelSEED	Microbial and plant metabolic modelling	Yes/yes	http://modelseed.org/
COBRAToolBox	Constraint-based modelling; MATLAB and Python	Yes/yes	http://opencobra.github.io/cobratoolbox/
OptFlux	Tool for metabolic engineering	Yes/yes	http://www.optflux.org/
